# The Impact of Central Venous Catheters on Pediatric Venous Thromboembolism

**DOI:** 10.3389/fped.2017.00005

**Published:** 2017-01-23

**Authors:** Julie Jaffray, Mary Bauman, Patti Massicotte

**Affiliations:** ^1^Children’s Hospital Los Angeles, University of Southern California Keck School of Medicine, Los Angeles, CA, USA; ^2^University of Alberta, Stollery Children’s Hospital, Edmonton, AB, Canada

**Keywords:** central venous catheter, pediatric, venous thromboembolism, venous thrombosis, thromboprophylaxis, tunneled line, peripherally inserted central catheter

## Abstract

The use of central venous catheters (CVCs) in children is escalating, which is likely linked to the increased incidence of pediatric venous thromboembolism (VTE). In order to better understand the specific risk factors associated with CVC-VTE in children, as well as available prevention methods, a literature review was performed. The overall incidence of CVC-VTE was found to range from 0 to 74%, depending on the patient population, CVC type, imaging modality, and study design. Throughout the available literature, there was not a consistent determination regarding whether a particular type of central line (tunneled vs. non-tunneled vs. peripherally inserted vs. implanted), catheter material, insertion technique, or insertion location lead to an increased VTE risk. The patient populations who were found to be most at risk for CVC-VTE were those with cancer, congenital heart disease, gastrointestinal failure, systemic infection, intensive care unit admission, or involved in a trauma. Both mechanical and pharmacological prophylactic techniques have been shown to be successful in preventing VTE in adult patients, but studies in children have yet to be performed or are underpowered. In order to better determine true CVC-VTE risk factors and best preventative techniques, an increase in large, prospective pediatric trials needs to be performed.

## Introduction

Central venous catheters (CVCs) are the single largest risk factor for pediatric venous thromboembolism (VTE) in all pediatric populations. The incidence of VTE in children is increasing, most likely secondary to advances in care of critically ill children and the increased insertion rate of CVCs (1). The use of CVCs has risen over the past decade due to their relative ease in placement and necessity for many lifesaving treatments, but this increase will likely lead to further escalating rates of pediatric VTE. CVCs can lead to VTE by causing vascular injury during insertion, as well as causing turbulent blood flow while the catheter is laying in the vessel lumen, with 85% of pediatric VTEs being CVC related (2, 3). Studies have been performed to evaluate catheter types, insertion locations, and catheter sizes that may lead to the highest probability of VTE. Most studies focus on limited patient populations, such as infants and patients in the intensive care setting or malignancy. Unfortunately, at this time, there are no guidelines in pediatric patients on how to choose the best central catheter type or insertion technique or prevention modalities to decrease the occurrence of VTE. CVC failures may be attributed to chemical or mechanical obstructions or VTE. This manuscript will review the current knowledge of risk factors regarding CVC-associated VTEs and thromboprophylactic interventions in order to provide the first steps toward creating guidelines to prevent thrombosis.

## Methods

### Search Strategy

We identified English articles using Medline (1975–August 2016) and Scopus (1975–August 2016). The search strategies comprised “venous thromboembolism,” “risk,” “children,” central venous line, central venous catheter, and venous access device, with multiple subject headings and text words per concept. Selectively exploding subject headings, with relevant subcategories, permitted ever-increasing specificity. We included systematic reviews, meta-analyses, randomized controlled trials, prospective cohorts, and retrospective cohorts. Case reports and case series were excluded.

### Study Selection

We excluded studies of patients older than 21 years based on the definition of pediatric age from the National Institute of Child Health and Human Development. For studies that included pediatric and adult patients, we excluded those without clear sub-analyses for patients under 21 years. Similarly, we excluded studies on arterial thromboembolism unless cases of VTE were included and clearly delineated in sub-analyses. Studies were identified as narrative reviews, commentaries, single case reports, retrospective case series, cross-sectional studies, case–control studies, cohort studies (retrospective and prospective), registry studies, or clinical trials, the first three of which were excluded.

This review included only pediatric CVC-related VTE with an aim to identify additional risk factors and populations at risk. An overview of the literature regarding different types and locations of CVCs, populations at risk, and prophylactic mechanisms will be discussed.

## Results

### CVC Data

Incidences of CVC-VTE in children reported in the literature vary largely, ranging between 0 and 74% for patients with CVCs (4). This variation is explained by differences in study design and characteristics of the study populations, such as age, underlying diseases, purpose of the CVCs, and use of prophylactic anticoagulation. Most importantly, reported incidences of CVC-VTE depend on clinical awareness and the diagnostic test used to investigate for VTE. Incidences of CVC-VTE in children identified through clinical diagnosis were 4–13% (5–7), through venous ultrasonographic screening were 1–44% (5), and screening *via* venography were 13–50% (6–8). Table 1 highlights CVC characteristics that may lead to an increased VTE rate.

**Table 1:** **Characteristics of CVC that may cause an increased incidence of VTE in children**.

CVC characteristic associated with increased VTE incidence	Reference
Externally tunneled CVCs (vs. internal CVCs, such as port-a-caths)	(9)
CVCs placed in the femoral vein (vs. upper extremity)	(10, 11)
CVCs placed on the upper left side, in the subclavian vein (vs. jugular), and percutaneous technique (vs. cut-down)	(11, 12)
Peripherally inserted central catheters (vs. tunneled lines)	(13)
Increased time CVC is in place, especially over 4 years	(14)
Multi-lumen CVC (vs. single lumen)	(15)
Polyurethane CVC material (vs. silicone estomer)	(16)
Blind approach technique for insertion (vs. ultrasound guidance)	(17)

*CVC, central venous catheter; VTE, venous thromboembolism*.

#### CVC Types and Insertion Locations

There are three distinct types of catheters: non-tunneled, which includes peripherally inserted central catheters (PICCs) and acute short-term lines; tunneled (Broviacs and Hickmans); and totally implanted CVCs (port-a-caths). The ideal type of CVC to minimize CVC-VTE is unknown. Although the populations described in systematic reviews and meta-analyses are heterogeneous in terms of patient location [e.g., neonatal or pediatric intensive care unit (ICU)] or underlying diagnoses (e.g., malignancy, sepsis), identification of additional risk factors (risk stratification) has not been carried out. As a result, determining the sole impact of the catheter type/location to the development of CVC-VTE is challenging. Furthermore, differing statistical analysis within studies of similar patient cohorts makes comparison of results across studies challenging. In a systematic review, 15,979 children with CVC were reviewed (18). Of all locations of CVCs implanted, the incidence of thrombosis was 1.7% [95% confidence interval (CI): 0.8–2.8]; however, the highest incidence was in umbilical CVCs 3.7% (CI: 0–12.2) and non-tunneled CVCs 3.7% (CI: 0–11.1). The lowest incidence of CVC-VTE was in tunneled lines [0.6% (CI: 0.2–1.2)] (18). In contrast, a meta-analysis by Vidal et al. (19), the frequency of thrombosis per 1,000 catheter days demonstrated PICCs, umbilical, and non-tunneled catheters as having the lowest frequency of CVC-VTE, 0.14–0.18, and tunneled catheters having the highest frequency, 0.28. The frequency of CVC-VTE was similar in the upper, 0.24 (CI: 0.17–0.31) and lower extremities, 0.2 (CI: 0.13–0.27) (19), although a trend toward increased CVC-VTE in femoral and subclavian insertion is reported with a recommendation for jugular vein insertion (11).

#### CVC Composition and Insertion Technique

There are varied catheter compositions, although polyurethane and silicone elastomer is typically used, with silicone catheters reported to be the least thrombogenic (16). There was no difference between antibiotic-impregnated or heparin-bonded catheters in the incidence of CVC-VTE (20). With respect to CVC diameter and patient age, there are conflicting relationships reported between jugular diameter and patient height, weight, age, and body surface area (21). The use of a 6Fr/2 mm CVC in patients <1 year of age was associated with complications in one study (22). Multiple lumen catheters are associated with increased occurrence of CVC-VTE, which may be due to the larger size of multi-lumen CVCs vs. a single lumen CVC (15, 23).

In terms of insertion technique among adults, ultrasound-guided approach vs. blind approach to line insertion resulted in decreased complications (17). Although not well defined, experts recommend ultrasound-guided approach to line insertion (24–26). In a pediatric study of patients with acute lymphoblastic leukemia, a percutaneous insertion technique was shown to have an increased incidence of CVC-VTE over cut-down technique (12).

### Patient Data

Venous thromboembolism in children can also occur as a secondary complication of severe primary diseases (Table 2). The most important exogenous risk factor is the presence of a CVC. Mahajerin et al. completed a systematic review and meta-analysis of risk factors and risk assessment models using case–control and non-case–control studies identifying five independent risk factors for VTE in pediatric hospitalized patients (27). The most significant risk factor for all hospital-acquired VTE (HA-VTE) was a CVC presence, followed by the following populations: those with systemic infection, ICU admission, mechanical ventilation, and prolonged hospital stay. HA-VTE was more prevalent in males than females (0.55) in non-case–control studies, which is consistent with the results found by Raffini et al. in the Pediatric Health Information System Data Base (1). Additionally, there is a bimodal age pattern in pediatric patients, revealing a peak incidence in neonates and then the adolescent age group. CVC-VTEs are more likely to occur in neonates, whereas non-CVC-VTEs are more likely to occur in adolescents (28). Populations reported to be at the highest risk for CVC-VTE include those with malignancy, sickle cell disease, congenital heart disease (CHD), chronic total parental nutrition (TPN) use, and trauma. Other patients shown to be at an increased risk for CVC-VTE are those with metabolic disorders, renal disorders such as nephrotic syndrome and kidney failure requiring dialysis, and those with cystic fibrosis (28–30). We will discuss in detail those at the highest risk for CVC-VTE.

**Table 2:** **Disease states that lead to an increased rate of CVC-associated venous thrombosis in children**.

Primary disease states with an increased VTE incidence
Malignancy with any type of CVC. For patients with a port-a-cath: increased risk in younger females with left-sided CVCs placed for a prolonged duration Neonates in an intensive care unit Critically ill children, especially those with a CVC-associated bloodstream infection or requiring mechanical ventilation Congenital heart disease Systemic infection Intestinal failure requiring total parental nutrition Trauma, especially those with a high injury severity score, received a blood product transfusion or an adolescent

*VTE, venous thromboembolism; CVC, central venous catheter*.

#### Children with Hematologic and Malignant Disease

The pathogenesis of thrombosis in patients with cancer is multifactorial. This includes the affect of the disease, in which tumor cells interfere with the hemostatic system by secreting procoagulant molecules and cytokines, as well as the invasion or compression of blood vessels my malignant cells (29). Chemotherapeutic agents are also highly thrombogenic, including asparaginase, which causes antithrombin deficiency and steroids which increase factor VIII/von Willebrand factor complex (30). However, the most important risk factor is the presence of a CVC, which is composed of thrombogenic material and obstructs venous flow and irritates the vessel wall (31). In a single-center study, children with malignancy and a port-a-cath, younger age, female sex, prolonged duration of a port-a-cath, and a left-sided device were independent risk factors for CVC-VTE (32).

Sickle cell disease has been demonstrated to have features associated with hypercoagulability, including increased levels of endothelial and platelet microparticles (33). Patients with sickle cell disease also have long-term CVCs, multiple hospitalizations, infections, and need for surgeries, thus increasing their risk for VTE. However, the reported incidence in retrospective studies for CVC-VTE is 0.2%, less than other high-risk populations (34, 35).

#### Critically Ill Neonates and Children

In a recent review, Park et al. reported an incidence of 9.2% of CVC-VTE in patients in the neonatal ICU (36). Alternatively, the rate of CVC-VTE was found to be 1.4 per 1,000 hospitalized neonates with CVC being an independent risk factor with a 0.9% risk (15). Fifty percent of children in PICUs have a CVC with a reported incidence of 0.8% symptomatic VTE (37). Children with CVC-VTE in the PICU had a median of 1 additional risk factor in addition to having a CVC (37), with catheter-associated blood stream infection being the most common presenting symptoms of CVC-VTE (38). Higgerson et al. in a prospective study with 11 pediatric ICUs identified other independent risk factors for thrombosis including mechanical ventilation and odds ratio (OR) 2.8 (CI: 0.98–7.93) (3).

#### Children with CHD

Children with CHD often have disruptions in the balance of hemostasis, which paradoxically could result in bleeding, thrombosis, or both. Cyanotic CHD is more commonly reported to have known hemostatic abnormalities compared with acyanotic CHD. Reported differences include abnormalities in coagulation proteins, platelets number and function, and red cell number and function altering hemostasis. These abnormalities can result in bleeding and/or thrombosis, with many having >1 abnormalities present (39). The reported incidence of symptomatic and asymptomatic CVC-VTE in children with CHD and CVC is 28% (40). Superior vena cava syndrome resulting from CVC-VTE is a serious consequence for children with CHD inhibiting further surgical palliation and may be life threatening.

#### Children with Systemic Infection

Systemic infection has been identified as an additional risk factor in all high-risk populations, although no studies have specifically evaluated systemic infection and CVC-VTE. However, in severe sepsis, dysregulation of the hemostatic system may lead to disseminated intravascular coagulation and result in micro-vascular thrombosis that may contribute to CVC-VTE (41). In addition, sepsis has been associated with the development of neutrophil extracellular traps (NETs), which are composed of extruded chromatin, which is negatively charge. The NETs are responsible for killing micro-organisms but have also been found to be highly prothrombotic (42). More studies in this area are needed.

#### Children with Intestinal Failure

One retrospective study reported 53 children with intestinal failure (43). Thirty subjects had venous imaging, and 57% of the imaged children had at least one symptomatic CVC-VTE with a mean of 5.6 ± 3.8 (range 1–12) CVCs per patient. CVC failure occurred in 53% of subjects, but there was not a significant difference in VTE rates in subjects who had a catheter occlusion or bloodstream infections and those that did not. By contrast, another study evaluating children on home TPN with inflammatory bowel disease were reported to only have an incidence of CVC-VTE of 10% (44).

#### Children after Traumatic Injury

The presence of a CVC continues to be the single greatest risk factor for VTE in pediatric trauma patients (OR 64, CI: 68.8–243.9) with a reported incidence of 0.2% for symptomatic CVC-VTE (45). Sixty-seven percent of VTE in trauma patients is at the site of the CVC (46). The combination of older age and increased injuries (increased injury severity score) escalate the risk of CVC-VTE (45). Consideration may be given to blood product transfusion, which is also reported to increase VTE in trauma patients (47). A recent study from the National Trauma Databank demonstrated that VTE risk increases in children beginning at age 13 and peaks at age 16, increasing to an incidence of 1% at age 16, independent of other VTE risk factors (47, 48).

### CVC-Related VTE Prevention

There are two main categories of prophylactic measures for children who are at risk for a CVC-VTE. These include mechanical prophylaxis, which consists of graduated compression stockings (GCSs) or intermittent pneumatic compression devices (IPCs), and pharmacological prophylaxis, such as systemic anticoagulation, ethanol locks, or fibrinolytics.

#### Mechanical Prophylaxis

Graduated compression stockings and IPCs improve venous blood return from the lower extremities by providing circumferential or intermittent pressure. Theoretically, the use of GCSs and IPCs may not prevent CVC-VTEs, especially in the upper extremity where many CVCs are placed, but there could be some benefit by improving overall venous blood flow and activating systemic fibrinolysis (49). Many children will not have access to mechanical prophylaxis due to size constraints of the devices. Unfortunately, studies have not been conducted in pediatric patients to determine if either modality is beneficial, but systematic reviews in adults have shown that both the use of GCSs or IPCs can prevent VTE (50, 51).

#### Thromboprophylaxis

Studies regarding the efficacy and safety of prophylactic anticoagulation in pediatric patients are limited. Meta-analysis on the use of antithrombotic agents (unfractionated heparin, low molecular weight heparin, warfarin, and antithrombin concentrate) and nitroglycerin did not demonstrate any significant efficacy with prophylaxis of CVC-VTE, although the studies were underpowered and closed early due to poor accrual (19).

#### Ethanol Locks

Ethanol lock therapy has been demonstrated to decrease the rate of central venous line-associated blood infections (CLABSI) in a number of pediatric populations with CVC. There are no anticoagulant properties associated with ethanol; however, ethanol decreases infection and there may be an interrelationship between bacteremia and CVC-VTE (52).

#### Lytic Locks

Tissue plasminogen activator (TPA) is the main fibrinolytic drug used for clot lysis and restoration or maintenance of catheter patency. A literature review focusing on pediatric patients with CVCs and the use of TPA revealed that 50–90% of catheters were cleared of thrombosis when TPA was instilled, with improved efficacy when doses where higher and dwell times were longer (53). Unfractionated heparin has also been used to maintain and improve catheter patency. A prospective cross-over controlled trial compared TPA to UFH in preventing CVC-VTE in patients receiving dialysis found TPA to be superior for VTE prevention (54).

## Summary

Venous thromboembolism is a serious and potentially life-threatening condition that has lead to increased morbidity and mortality in pediatric patients. CVCs remain a predominant risk factor for VTE in children, and their use and rate of insertion continue to climb. Data evaluating the incidence and specific risk factors for CVC-VTE with various CVC types and medical conditions are limited. Most studies are predominantly retrospective, single institution, and focused on a single central catheter type. This article sought to provide a brief review of the literature regarding pediatric CVC-related VTE in order to highlight various risk factors linked to catheter characteristics and the patients’ medical history.

The overall incidence of CVC-VTE in pediatrics varies greatly due to differences in patient population, catheter type, detection methods (screening vs. requiring clinical symptoms), and imaging modality. CVC type and its effect on VTE incidence remain the most significant question. PICCs, which are usually placed into vessels of smaller caliber with a longer intravascular course, are being placed even more readily than other forms of CVCs (55). Therefore, truly understanding the VTE risk associated with PICCs vs. other CVCs is of great importance. Although the study results vary, increased CVC-VTE incidence has been found with externally tunneled CVCs over implanted CVCs, PICCs and umbilical lines over tunneled lines, CVCs placed in the subclavian and femoral vein, lines placed in the upper left side, multi-lumen CVCs, lines inserted without ultrasound guidance, and CVCs made from polyurethane over silicone.

Various patient populations have been shown to have an increased risk of VTE. There is difficulty determining if these populations are more at risk for VTE overall or CVC-related VTE specifically. Children with the highest risk of CVC-VTE are those with malignancy, systemic infection, CHD, gastrointestinal failure, sickle cell disease, those in an ICU, and those with a traumatic injury. Besides having a CVC, some of these patients have many other compounding risk factors, such as being in an inflammatory state, having decreased mobility, and being exposed to thrombogenic medications such as steroids or asparaginase.

Preventative measures, such as mechanical or pharmacological prophylaxis, have not been largely studied in pediatric patients, and thus, their utility in CVC-VTE prevention is unknown. Recurrent TPA locks have been shown to improve catheter flow in patients with CVCs obstructed by thrombosis.

In conclusion, our review illustrates the need for large prospective pediatric studies to truly evaluate catheter types, insertion techniques, and clinical characteristics to create guidelines for our patients prior to receiving a CVC. We hope that this review of the current literature will lead to a better understanding of the risk factors linked with CVC-VTE, which will promote the needed prospective evaluation.

## Author Contributions

JJ assisted in writing and editing the manuscript. MB assisted in performing the review and writing the manuscript. PM assisted in performing the review and writing and editing the manuscript. JJ and PM addressed the reviewer comments.

## Conflict of Interest Statement

The authors declare that the research was conducted in the absence of any commercial or financial relationships that could be construed as a potential conflict of interest.
